# Endometrial Histology of Depomedroxyprogesterone Acetate Users: A Pilot Study

**DOI:** 10.1155/IDOG/2006/69402

**Published:** 2006-02-23

**Authors:** Andrea Ries Thurman, David E. Soper

**Affiliations:** Department of Obstetrics and Gynecology, Medical University of South Carolina, 96 Jonathan Lucas Street, CSB #628B, PO Box 250619, Charleston, SC 29425, USA

## Abstract

*Objective*. To obtain pilot data on the endometrial histology of Depomedroxyprogesterone acetate (Depo-Provera, DMPA) users experiencing breakthrough bleeding (BTB) versus users with amenorrhea. To compare the endometrial histology of patients who used DMPA continuously for 3–12 months versus those who used it for 13 months or more. *Methods*. Cross-sectional study. Endometrial biopsy was obtained fromall consenting patients who used DMPA for at least 3 months. Patients were divided into those with BTB in the last 3 months versus those with amenorrhea for at least 3 months. Histology results and duration of therapy were compared. *Results*. The proportion of women with chronic endometritis, uterine polyps, atrophic, proliferative, or progesteronedominant endometrium did not differ between those DMPA users with BTB versus those with amenorrhea. Duration of therapy did not correlate with symptoms of BTB or endometrial histology. Chronic endometritis was the most common histologic finding (10/40, 25%) and occurred more often in women experiencing BTB (35% versus 15%) (RR 1.62 CI 0.91–2.87). Moreover, 45% of women with BTB had received DMPA for more than 12 months. *Conclusions*. BTB was more common than previously reported in women using DMPA for more than 12 months. Chronic endometritis, which may indicate an underlying infectious or intracavitary anatomic etiology, has not been previously reported as a frequent finding in DMPA users, and may be related to ethnic or other sociodemographic characteristics of our patient population. Further study to elucidate the etiology of chronic endometritis in these patients is warranted.

## INTRODUCTION

Depomedroxyprogesterone acetate (DMPA or Depo-Provera) is a long-acting injectable contraceptive, approved for use in the United States in 1992 [1–3]. DMPA has been available worldwide since the 1960s and has been used by more than 30 million women in more than 90 countries [1–3]. This convenient contraceptive has typical failure rates that are lower than all the other temporary, hormonal methods and a theoretical failure rate which is lower than surgical sterilization [1–3]. The most common side effect of DMPA is breakthrough bleeding (BTB), which occurs in 70% of patients in the first year, and in approximately 10% thereafter [1]. Unfortunately, 25% of patients discontinue this effective contraceptive in the first year because the bleeding is intolerable [4]. Therapy for irregular bleeding on DMPA has had limited success [5, 6]. Treatment with estrogen, nonsteroidal antiinflammatory drugs (NSAIDs), or more frequent DMPA injections has been recommended by various authors, with some of these recommendations based on treatment trials of Norplant users and, more often, on assumptions regarding the endometrial histology of DMPA users [1, 2, 5–11]. Physicians must often resort to reassuring patients that irregular bleeding is an expected side effect, which will abate with time. It is likely that if the underlying endometrial histology in patients suffering from this side effect was known, therapy could be more directed and successful. The goal of this study is to describe the endometrial histology of DMPA users who are amenorrheic versus those who experience BTB and to obtain data on the endometrial histology based on duration of continuous DMPA use.

## METHODS

This is a cross-sectional study. Based on previous studies, we predicted that 75% of amenorrheic DMPA users would have a progesterone-dominant or atrophic endometrium compared to only 25% of patients with BTB on DMPA [12, 13]. Based on these assumptions, 18 patients were required in each group to have an 80% power to detect a 50% difference with an alpha of 0.05. This power analysis was calculated using Epi Info 2002, CDC Software, Atlanta, Ga.

All patients presenting to the outpatient gynecology clinic who used DMPA for at least 3 months were asked to participate in the study. We recruited the first 20 patients with BTB and the first 20 patients with amenorrhea on DMPA. This study was approved by the Institutional Review Board at the Medical University of South Carolina.

Inclusion criteria were the ability to give informed consent and use of Depo-Provera continuously for at least 3 months. BTB was defined as unscheduled uterine bleeding in the last 3 months. Amenorrhea was defined as no uterine bleeding for at least 3 months. Patients were excluded if they had other possible causes of vaginal bleeding: using other hormone medications in the last 3 months, intrauterine device use, and active cervical dysplasia greater than lowgrade squamous intraepithelial neoplasia. Patients were also excluded if they had an absolute or relative contraindication to endometrial biopsy including pelvic inflammatory disease, diabetes, active cervical infection, blood dyscrasias, and pregnancy.

Informed consent was obtained by the physician performing the biopsy (AT). The duration of uninterrupted DMPA use, in months, and the patient's age, race, gravidity, and parity were recorded. The patient's pap smear and cervical tests for *N gonorrhoea* and *Chlamydia trachomatis* within the last year were confirmed to be negative prior to the procedure. A wet prep was prepared to determine the presence of yeast vaginitis or bacterial vaginosis (BV). BV was diagnosed using Amsel's criteria (14). An endometrial biopsy was performed, using a 3 mm Unimar Pipelle (Wilton, Conn).

A pathologist, who was blinded to the patient's clinical symptoms, interpreted the endometrial biopsies. Chronic endometritis was defined as greater than or equal to 2 plasma cells in the endometrial sample. Treatment regimens for BTB were not investigated in this study.

Chi-square statistic and 2-tailed Fisher's exact test were used to compare the proportions of patients with various endometrial histologies in each group.

## RESULTS

The biopsy results of patients experiencing BTB on DMPA (*n* = 20) are presented in Table 1. The endometrial histology results of patients with amenorrhea on DMPA (*n* = 20) are presented in Table 2. The comparison of histologic diagnoses among women with BTB versus women with amenorrhea are shown in Table 3. Chronic endometritis was more than twice as common in DMPA users complaining of BTB (7/20, 35% versus 3/20, 15%) (RR 1.62 CI 0.91–2.87). The significance of this finding is limited by sample size. An atrophic, progesterone-dominant, or no endometrium was more commonly found in those with amenorrhea (RR 0.50 CI 0.23–1.10).

Because the DMPA package insert shows a significant decrease in BTB at the 1-year mark, we also compared women who used DMPA continuously for 3–12 months (*n* = 19) with women who received DMPA continuously for 13 months or longer (*n* = 21) in Table 4. Surprisingly, there was no difference between the incidence of BTB among women who had been on DMPA for less or more than 1 year, but the analysis is limited by sample size. Also, despite the assumption that women trend to a progesterone-dominant endometrium over time, there was no difference in various histologic diagnoses between women on DMPA for more or less than 1 year. There was no difference in the rate of BV among patients with BTB (5/20) versus those with amenorrhea (7/20) *P* = .49. Also, there was no difference in the incidence of BV in patients with chronic endometritis (4/10) versus those without chronic endometritis (8/30) *P* = .45, but these data are limited by sample size. We did not collect information on body mass index (BMI).

Finally, a very unusual finding was a patient with BTB who had a placental site nodule. She had been on DMPA for 3 months postpartum, after an uncomplicated vaginal delivery, and her beta human chorionic gonadotropin level was zero. Her BTB resolved within 1 month of biopsy, and no further treatment was necessary.

## DISCUSSION

This study found that women using DMPA exhibit a variety of endometrial histologic findings. Chronic endometritis was a frequent finding in our patients, which has not been previously reported. Using various combinations of the key words—Depomedroxyprogesterone acetate, bleeding, injectable contraception, side effects, endometrium, and histology—we located 4 studies on the endometrial histology of DMPA users [12–15]. Mishell et al and Karim and colleagues performed sequential endometrial biopsies on postpartum women given DMPA, the majority of whom were Caucasian or Middle Eastern, and found that atrophic or inactive endometriums prevailed with duration of DMPA use [14, 15]. The incidence of BTB decreased with time, but neither group correlated endometrial histology with patient symptoms [14, 15]. Lee performed sequential endometrial biopsies on 14 patients given DMPA postpartum and found that patients with BTB tended to have a proliferative endometrium while patients with amenorrhea had an atrophic endometrium [12]. Jeppsson obtained endometrial biopsies on 11 Swedish DMPA users, all of whom were amenorrheic and had been using DMPA continuously for a mean of 36 months [13]. All of these patients exhibited marked endometrial atrophy [13].

We found chronic endometritis in 7 women with BTB (35%) and 3 women with amenorrhea on DMPA (15%). Chronic endometritis was not reported in previous studies of DMPA patients and may reflect ethnic or other demographic factors of our study population, as BTB on DMPA has been correlated with ethnicity [16]. Chronic endometritis has also not been found in studies of the endometrial histology of Mircette, Cyclofem, or Norplant users [17–19]. In a review of endometrial bleeding, Ferenczy postulates that some endometrial bleeding is the result of an inflammatory environment, caused by endometrial atrophy, friction between the endometrial surfaces, and subsequent microerosions [20]. Chronic endometritis may also arise from intracavitary lesions such as submucosal fibroids or uterine polyps. None of our patients had clinical evidence of an active cervical infection, which suggests that the chronic endometritis found in this study was inflammatory rather than infectious. Our data is limited by the fact that we relied on a negative screening test for chlamydia and gonorrhea within the year and endometrial cultures were not performed. Chronic endometritis has been used as a surrogate for the diagnosis of atypical pelvic inflammatory disease [21]. In our patient population, at risk for sexually transmitted infections, further investigation regarding the etiology of chronic endometritis is warranted.

Our findings suggest that antimicrobial or antiinflammatory therapy may be the most effective to treat BTB in these patients. This new avenue of therapy for BTB in DMPA users needs to be investigated in future studies. The treatment of BTB on DMPA is based on assumptions of the endometrial histology, and many of the recommendations are from data of Norplant users with BTB [9, 10, 22]. Empiric treatment of BTB on DMPA has had limited success [5, 6]. Estrogen therapy is recommended when it is suspected that the patient has developed a fragile, atrophic endometrium [1–3, 5, 6, 11]. More frequent DMPA injections have been advocated, but Harel and coworkers found that the bleeding patterns of adolescents who received DMPA every 6 weeks were similar to the patterns of those who received DMPA every 12 weeks [8]. Progesterone-dominant oral contraceptives have also been suggested to treat BTB on DMPA, which assumes the patient has a proliferative endometrium which needs to be converted to an atrophic or progesterone effect pattern [1, 8]. NSAIDs have also been advocated for BTB in DMPA users, based on treatment studies of Norplant users [9, 10]. NSAIDs can decrease endogenous prostaglandins associated with BTB.

The package insert for DMPA reports that the level of medroxyprogesterone acetate (MPA) should be 1–7 ng/mL at 3 weeks after the DMPA injection and < 100 pg/mL at 120–200 days postinjection. A study of Thai patients, whose average BMI was 22, found that estradiol and progesterone levels were not correlated with the presence or duration of BTB in DMPA users [23]. The serum levels of progesterone after DMPA administration are high enough that contraceptive efficacy is not influenced by the patient's weight [6]. However, DMPA does not suppress follicle stimulating hormone, and therefore estrogen levels are comparable to the early follicular phase and will be influenced by the patient's weight [6]. It is possible that BMI is associated with BTB, and this should be an area of future research.

It has been shown that adolescents who transition from combined oral contraceptives to DMPA have less BTB than those who begin DMPA without prior hormone exposure [8]. It is presumed that upregulation of endometrial progesterone receptors by estrogen in oral contraceptives account for this finding [8]. A previous study found that endometrial glandular progesterone receptor immunoreactivity was similar in patients experiencing bleeding or amenorrhea on DMPA [23]. However, the immunoreactivity of progesterone receptors in the endometrial stroma was elevated in patients with amenorrhea on DMPA, as compared to those with bleeding on DMPA [23]. However, among patients with bleeding on DMPA, the duration of bleeding did not correlate with progesterone receptor quantity, indicating that additional processes, namely, inflammatory or infectious, may be contributing to the bleeding [23].

Our pilot study was limited by the small number of patients. Our pretest power analysis, based on commonly applied assumptions regarding the endometrial histology of DMPA users, was inaccurate. We were surprised to find that less than 25% of our patients had evidence of a progesteronedominant endometrium and so many of the patients had evidence of chronic endometritis. We would require additional 134 patients to render a statistically valid evaluation of chronic endometritis. Finally, the cross sectional design of the study limited analysis of how the endometrial lining changes with time in DMPA users.

In summary, this study found that DMPA users can exhibit several endometrial histologic diagnoses, and the tissue findings do not correlate easily with the patient's symptoms or duration of medication use. Chronic endometritis was the most common histologic diagnosis in women experiencing BTB and has not previously been reported. Chronic endometritis likely reflects an underlying infectious or anatomic abnormality which would completely change the management of BTB in these patients, and will require further evaluation.We believe further studies to elucidate the etiology of BTB in DMPA users are indicated.

## Figures and Tables

**Table 1:** Endometrial histology in patients with breakthrough bleeding on DMPA.

Age (y)	Race (A = African	Gravidity/parity	Months of continuous	Endometrial
	American, C = Caucasian)		DMPA use	histology

37	A	3/1	62	Polyp
42	A	3/2	60	Benign inactive
34	A	3/3	42	Benign inactive
20	C	1/1	04	Chronic endometritis
35	A	4/4	03	Chronic endometritis
25	A	1/1	06	Proliferative
21	A	1/1	03	Placental site nodule
33	C	2/1	06	Progesterone dominant
26	A	1/1	48	Progesterone dominant
28	A	2/2	24	Chronic endometritis
29	A	1/1	09	Atrophic
24	C	3/2	12	Atrophic
30	C	3/2	48	Atrophic
39	A	4/4	120	Chronic endometritis
24	A	1/1	36	Chronic endometritis
30	A	8/6	03	Chronic endometritis
21	A	1/1	18	Polyp
43	A	1/1	09	Menstrual endometrium
21	A	1/1	09	Chronic endometritis
19	A	1/1	03	Menstrual endometrium

**Table 2:** Endometrial histology in patients with amenorrhea on Depo-Provera.

Age (y)	Race (A = African	Gravidity/parity	Months of continuous	Endometrial
	American, C = Caucasian)		DMPA use	histology

48	A	4/4	48	Atrophic
39	A	4/2	03	No endometrium
23	C	0/0	15	Benign inactive
21	A	0/0	36	Chronic endometritis
37	A	0/0	26	Polyp
28	A	4/4	12	Benign inactive
32	A	3/1	15	Polyp & proliferative
38	A	1/1	36	No endometrium
27	C	1/1	18	Benign inactive
38	C	1/1	60	Progesterone dominant
22	A	1/1	12	Atrophic
36	A	6/1	09	Progesterone dominant
18	A	0/0	03	Chronic endometritis
18	A	1/1	03	Atrophic
19	A	0/0	03	Progesterone dominant
33	A	1/1	12	Atrophic
31	A	2/2	60	Progesterone dominant
29	A	3/2	27	Progesterone dominant
25	C	1/0	60	Proliferative
26	A	3/1	24	Chronic endometritis

**Table 3:** Comparison of endometrial histology among women with breakthrough bleeding (BTB) versus women with amenorrhea.

Endometrial	Patients with BTB	Patients with amenorrhea	Chi-square *P*	Fisher's exact
histology	(*n* = 20)	(*n* = 20)	value	2-tailed test *P* value

Chronic endometritis	7	3	.15	N/A
Atrophic or no endometrium	3	6	N/A	.45
Proliferative	1	1	N/A	1.00
Progesterone dominant	2	5	N/A	.40
Polyp	2	2	N/A	1.00
Benign inactive	2	3	N/A	1.00
Menstrual	2	0	N/A	.49
Placental site nodule	1	0	N/A	1.00

**Table 4:** Comparison on symptoms and endometrial histology among women using Depo-Provera (DMPA) for 3–12 months versus those using it for 13 or more months.

Symptom or	DMPA Use 3–12	DMPA Use 13 or more	Chi-square *P*	Fisher's exact
endometrial histology	months (*n* = 19)	months (*n* = 21)	value	2-tailed test *P* value

Breakthrough bleeding	11	9	.35	N/A
Chronic endometritis	5	5	N/A	1.00
Atrophic or no endometrium	6	3	N/A	.26
Proliferative	1	1	N/A	1.00
Progesterone dominant	3	4	N/A	1.00
Polyp	0	4	N/A	.11
Benign inactive	1	4	N/A	.35
Menstrual	2	0	N/A	.22
Placental site nodule	1	0	N/A	.48
